# Supplementary material for: Are carbon emissions trading and green financial instruments synergistic? -Comprehensive quantitative research based on content analysis
Source: PLoS One. 2024 Mar 7;19(3):e0298601. doi: 10.1371/journal.pone.0298601 (PMC10919601; doi:10.1371/journal.pone.0298601)
Supplement: S1 Data — (ZIP) [file pone.0298601.s001.zip › Data/地级市Z_F工作报告.zip!/╔╜╬≈/┴┘╖┌╩╨/└┤╫╘╣1⁄2╓┌║┼í╛┬φ┐╦╩2╛▌═°í┐_2010.docx]

各位代表：

　　现在，我代表市人民政府向大会报告工作，请予审议，并请市政协委员和其他列席人员提出意见。

　　 一、2009年工作回顾

　　2009年，是新世纪以来我市经济社会发展遇到困难最多的一年。一年来，在中共临汾市委的坚强领导下，在市人大、市政协的大力支持下，我们坚持以科学发展观为统领，团结和带领全市人民，全力应对国际金融危机冲击，统筹推进"保增长、保民生、保稳定"各项工作，转型发展、安全发展、和谐发展迈出坚实步伐，经济和社会各项事业取得了新的成绩。

　　——经济实现平稳增长。面对严峻形势，我们千方百计促进经济增长，实现了经济增速转负为正、回升向好的目标。全市生产总值完成763.1亿元，按可比价格计算，增长4.2%；规模以上工业增加值完成372.6亿元，按可比价格计算，增长3.04%；固定资产投资完成419亿元，增长58.1%；社会消费品零售总额完成272.6亿元，增长21.7%；财政总收入达到137.7亿元，完成省政府下达的调控任务；一般预算收入完成62.9亿元，增长16%；城镇居民人均可支配收入达到12247元，增长9.3%；农民人均纯收入达到4749元，增长8.1%；居民消费价格指数为100.7%。

　　——项目建设成效显著。投资额创历史新高，争取中央和省级扩大内需项目资金14.35亿元，全市在建项目固定资产投资达到千亿元以上。其中，重点监控的209个项目开工建设190个，竣工83个，完成投资225亿元。交通建设全面推进，临吉高速公路、临午线、桃临线完成投资32.1亿元，263公里沿黄旅游扶贫公路全线贯通；乔李机场复航改造项目上报国家发改委审批；青兰-京昆高速联络线、城区北环高速、中南部铁路出海通道、大西高速铁路客运专线启动建设，全市新增公路通车里程514公 里。城市基础设施建设得到加强，市区"两桥十街"工程，已有锣鼓大桥、西北环、广宣街等9项完工，五一路立交桥开工建设；滨河西路北延工程基本完工，西环、西南环和临汾一级客运站工程建设进展顺利。重大民生项目按计划有序实施，汾河城区段生态治理工程河道治理已经完成，生态建设全面启动；临汾新高中、新医院开工建设，市妇幼保健院综合大楼投入使用。

　　——结构调整扎实推进。传统产业优化升级，煤矿兼并重组取得阶段性成果，392座煤矿中312座签订了交易协议，182座煤矿"六长"到位。新规划的129座煤矿已有112座办结采矿许可证。山焦150万吨焦炉扩建、临钢中板生产线改造、兆光二期2×60万千瓦等项目竣工投产。新兴产业不断壮大，华翔美的35万吨精密铸件、平阳重工液压支架、乡宁戎子葡萄酒、同世达20万吨甲醇等一批项目进展顺利。侯马开发区高新技术孵化中心开工建设，光宇LED新电源、洪洞双银电热膜等高新技术项目取得新的突破。第三产业健康发展，旅游业实现综合收入85亿元，增长20%。侯马方略保税物流中心配套工程完工。博浩园馨城、恒安新东城等一批房地产项目陆续竣工。

　　——"三农"工作稳步发展。各项惠农政策全部落实。各级财政用于农林水的支出达到12亿元，增长13.9%。农业综合生产能力得到提高，建设粮食丰产方37个，改造中低产田26万亩，实施水保治理120万亩。严格保护农业用地，耕地占补基本平衡。和川引水工程开闸蓄水，10座水库完成除险加固。粮食总产达到16.68亿公斤，水果、蔬菜及畜产量持续增加，优质农产品比重上升。新建和扩建农业产业化龙头企业25户，发展农民专业合作社953个。中国(山西)特色农产品交易博览会引资28.2亿元。完成培训农民30万人(次)，转移农村劳动力3.5万人。新农村建设县域总体规划编制全部完成，234个重点推进村基础设施得到改善，连片区发展到81个，新增沼气用户2万多户。扶贫开发完成整村推进32个村，移民搬迁5900人。"五个全覆盖"超额完成年度计划。

　　——生态环境明显改善。加强工业污染治理，否决50多个不符合环保政策的项目，淘汰33户污染企业，97户重点企业实现在线监控。强化生活污染治理，新增城市集中供热面积409万平方米，完成煤气、天然气扩户2万户，建成污水和垃圾处理厂12个。开展城乡绿化，完成造林78万亩，森林覆盖率达到31.4%，新增城市绿化面积248.6万平方米。17个县（市、区）二级以上天气全部超过310天，其中，市区二级以上天气达到334天，空气综合污染指数下降到1.72。全市二氧化硫、化学需氧量分别减排1900吨和1050吨。汾河临汾段化学需氧量和氨氮浓度分别下降67%和19.9%，地表水质进一步改善。节能减排取得明显成效，万元生产总值综合能耗下降6.6%。

　　——安全生产形势好转。政府监管责任和企业主体责任进一步落实，煤矿5人小组监管制度全面实施。扎实开展了以煤矿、非煤矿山、尾矿库为主的各行各业安全专项整治，取缔非煤矿山企业632户、尾矿库116座，排查治理各类安全隐患9000余条。打击非法违法开采"风暴"行动取得阶段性成效，抓捕非法涉矿人员262人，追究刑事责任42人，处理监管责任人员33人，兑现举报奖励28.6万元，私采滥挖现象得到有效控制。全市工矿企业事故下降41.4%，各类安全生产事故死亡人数下降46.1%，煤炭百万吨死亡率比省控指标低0.73，基本遏制了事故频发的势头。

　 ——社会管理力度加大。整顿和规范市场秩序，开展了食品药品安全、非法传销、农资打假等专项整治行动，查处各类案件3426件。加大价格监督检查力度，对236个品种进行了常规价格监测，确保了市场价格稳定。妥善处置洪洞猪蓝耳病、壶口冰凌等突发事件，有效防控了甲型H1N1流感。信访工作得到加强，赴省和进京上访人次分别下降15.3%和21.1%。深入开展"平安临汾"创建活动，实施了打击"两抢一盗"和"黄赌毒"专项行动，刑事犯罪和社会治安案件分别下降17.2%和10.7%。

　　——民生事业不断进步。在财政十分困难的情况下，坚持优化支出结构，规范行政开支，压缩一般性公务支出13%，压减会议经费24%，保证公教人员工资发放，集中财力支持民生事业。全市一般预算支出131亿元，涉及民生支出占到33.7%。加大财政融资力度，共融资38.8亿元，保障重点民生工程的实施。教育工作成效显著。义务教育阶段经费保障机制全面落实，各级财政用于教育的投入近25亿元，增长17.2%。办学条件进一步改善，完成中小学校舍改造42.1万平方米，新建和改扩建中小学443所。教育管理得到加强，基础教育质量明显提高，职业教育、高等教育和民办教育健康发展。医疗卫生水平不断提升。各级财政用于医疗卫生的投入10.1亿元，增长32.3%。城市社区卫生机构、乡镇卫生院和村级卫生室建设扎实推进，10个县级综合医院达到二级乙等以上水平，市人民医院晋升"三甲"医院。新农合参保人数达到289万人，参合率90.4%，报销参保患者医药费2.8亿多元。社会保障逐步完善。社会保险扩面征缴超额完成省定任务，退休人员养老金全部及时足额发放。21.1万城乡低保对象和1.1万农村五保供养对象实现应保尽保。采取"两抗四保"措施，成功抗击暴雪灾害。补贴市区居民集中供热、供气2.6亿元。投入7.3亿元，确保了临纺等5户企业政策性破产顺利推进。创业就业力度加大。新增城镇就业4.2万人，实现下岗失业人员再就业9785人。市县两级争取资金1.13亿元购买公益性岗位，解决了4060名困难群众的就业问题。通过创业实现就业1.08万人。住房安居工程顺利实施。建设经济适用房31万平方米、5225套，廉租住房23万平方米、4625套，实施城市棚户区改造3万平方米，完成农村困难群众住房改造1200户。科技文化体育等事业全面发展。科技创新力度不断加大，文化广播电视事业日益繁荣，群众性体育活动广泛开展，统计和人口计生工作受到上级表彰。国防动员和后备力量建设扎实推进，"双拥"工作成效显著。民族宗教、文物、人防、外事、侨务、方志、档案、气象、测绘、地震、老龄、残疾人、慈善救助和红十字等工作取得了新的成绩。表大会第六次会议上 临汾市市长 罗清宇）——民主法制和精神文明建设得到加强。自觉接受人大法律监督和政协民主监督，认真落实市人大"六项重点工程"和煤炭资源整合督查意见，政府相关部门整改了评议提出的问题。支持各民主党派、工商联、无党派人士参政议政，办理人大代表建议和政协委员提案493件，办复率达到100%。"法治临汾"建设取得实效，普法工作受到国家级表彰。群团组织作用得到充分发挥。基层民主制度不断完善。政府自身建设得到加强，党风廉政建设责任制有效落实，煤焦领域反腐败取得阶段性成果。监察和审计工作成效显著，执法监察、项目督查扎实推进，农村低保、优抚基金专项清理成效突出，公路"三乱"得到明显遏制，行风政风进一步好转，行政审批和财政体制改革逐步深化。深入开展了"爱我临汾、树我形象、建我家乡"活动，精神文明创建取得丰硕成果，我市荣获"山西省未成年人思想道德建设先进市"称号，侯马、翼城、古县、安泽被评为"省级文明和谐城市"。

　　各位代表，在这里我向大会郑重报告，2009年在极度困难的情况下，我市经济实现了平稳增长，民生事业快速发展，市政府向全市人民承诺的10件实事全部完成年度目标。这些成绩的取得，是中共临汾市委正确领导的结果，是市人大、市政协鼎力支持的结果，是全市各级干部和广大人民群众同心同德、顽强拼搏的结果。在此，我代表市人民政府，向辛勤工作在各条战线上的工人、农民、知识分子和广大干部群众，向为临汾发展贡献智慧和力量的人大代表、政协委员、各民主党派、工商联和无党派人士，向为临汾建设发挥余热的离退休老同志，向关心支持临汾发展的驻临部队指战员、武警官兵，向积极参与临汾经济社会建设的专家、学者、企业家以及社会各界人士，致以崇高的敬意和衷心的感谢!

　　各位代表，成绩来之不易，经验弥足珍贵。一年来的实践充分证明，只要始终坚持以科学发展观为统领，认真贯彻落实党中央、国务院、省委、省政府的决策部署，我们就能够沿着正确的轨道不断前进；只要始终坚持依靠广大干部群众，凝聚社会方方面面的智慧和力量，我们就一定能够克服一切困难，开创经济社会发展的新局面；只要始终坚持求真务实、真抓实干，创造性地开展工作，我们就一定能够重塑临汾新形象、再铸临汾新辉煌!

　　在看到成绩的同时，我们也清醒地认识到存在的问题和差距。主要是经济结构不合理，发展方式落后，可持续发展能力不强；安全与发展的矛盾仍然没有彻底解决，安全生产水平仍需提高；城乡基础设施建设滞后，城镇化水平依然较低；教育、医疗等民生事业欠账较多，环境状况与人民群众的要求还有差距；一些部门和干部服务意识不强、办事效率不高，政府自身建设有待加强。对此，我们将高度重视，采取更加有效的措施认真加以解决。

　　二、2010年主要任务

　　今年是本届政府任期的最后一年，也是巩固经济回升向好势头、努力完成"十一五"规划的关键一年。做好今年的工作，责任重大，意义深远。

　　总体上看，今年的经济发展环境要好于去年。世界经济开始复苏，国内经济持续向好，这是我们谋取新发展的先决条件；中央保持宏观经济政策的连续性和稳定性，省委、省政府继续扩大投资、改善民生，这是我们创造新业绩的坚强支撑；市委确定的好思路、好目标和全市上下形成的好氛围、好势头，这是我们再铸新辉煌的有力保障。但是，国际金融危机的后滞效应远未消除，国内经济持续向好的基础仍不牢固，我市煤炭产业的规模效益发挥需要一个过程，焦化、冶金行业兼并重组在短期内会影响经济增速，新兴产业尚未形成竞争优势。我们既要看到有利条件，又要看到不确定因素，更加周密地做好应对困难和挑战的准备，牢牢把握工作的主动权。

　　2010年政府工作的总体要求是：以邓小平理论和"三个代表"重要思想为指导，深入贯彻落实科学发展观，按照《临汾科学发展规划纲要》的总体部署，紧紧围绕经济平稳较快增长这一首要任务，以转变经济发展方式为主线，以保障和改善民生为目标，努力做好调节经济运行、优化产业结构、夯实"三农"基础、建设特色城市、改善生态环境、提升安全水平、坚持改革开放、构建和谐社会八项工作，加快转型发展、安全发展、和谐发展，建设经济繁荣、社会和谐、民生殷实、环境优美的新临汾。

　　今年经济社会发展的指导性指标是：地区生产总值增长8%；全社会固定资产投资增长20%；社会消费品零售总额增长16%；进出口总额增长5%；财政总收入和一般预算收入均增长7%；城镇居民人均可支配收入和农民人均纯收入均增长9%；居民消费价格综合水平涨幅控制在3%左右。约束性指标是：万元地区生产总值综合能耗下降4.6%；二氧化硫、化学需氧量排放分别下降2%和3%；城镇登记失业率控制在4.3%以内。

　　实现上述目标，必须突出重点，抓住关键，扎实做好八个方面的工作：

　　（一）强化经济调节，确保平稳较快增长

　　保持经济平稳较快增长仍然是今年工作的首要任务。我们将以项目建设为重点，发挥源头产业优势，创优发展环境，全力促进经济快速发展。

　　大力推进项目建设。进一步扩大投资规模，千方百计争取国家、省投资支持，创造条件吸引民间资本参与。突出抓好216个重点建设项目，总投资1004.2亿元，当年完成投资387.1亿元。一是实施106个产业项目。总投资676.9亿元，当年完成投资221.4亿元。大唐国际河西电厂2×30万千瓦、霍州发电厂2×60万千瓦和浮山中强煤电一体化等项目完成投资计划，平阳重工新增2000台高端液压支架、蒲县500万吨洗煤等项目竣工投产。二 是实施79个基础设施项目。总投资291.5亿元，当年完成投资145.8亿元。重点推进5条铁路、7条高等级公路建设和乔李机场复航改造项目。开工建设投资18亿元的张台铁路，配合抓好大西高铁、中南部出海通道建设和南同蒲电气化、侯西复线改造；做好长临高速、霍永高速和霍侯路改造项目前期工作，争取今年开工；加快临吉高速、市区北环高速、京昆-青兰联络线和临汾一级客运站建设，全面完成临午线、河西南北大道、五一路立交桥、大运高速土门连接线等重点工程，完成2000公里县乡村公路联网通达，启动实施"村连村、户户通"工程。三是实施31个社会事业项目。总投资35.8亿元，当年完成投资19.9亿元。加快晋国遗址博物馆建设，临汾新高中、新医院建设完成主体工程。启动建设临汾市应急指挥中心、市博物馆和市精神病医院。切实保障项目用地，用足用活土地政策，确保项目落地。多渠道筹措项目资金，保证项目顺利推进。全面加强项目管理，确保工程质量和工程进度。

　　强化源头产业支撑。引深煤炭资源整合，尽快办结规划矿井所有手续，主体企业管理团队全部到位，妥善处理各方利益关系，兑现被兼并煤矿的补偿资金；对改扩建和新建矿井，抓紧完成地质报告和初步设计，确保上半年全部开工。加快煤矿复工复产步伐，对过渡性矿井和单独保留矿井，明确时限，严格标准，在确保安全的前提下，尽快恢复生产，力争原煤产量达到4000万吨以上。同时，加快非煤矿山企业复工复产步伐，努力增加铁矿石、石灰石等产品产量，保证下游企业原料供应。

　 创优企业发展环境。深入企业搞好服务，搭建原料供求、市场营销和银企合作平台，解决企业在生产经营中遇到的困难。强化县域之间、企业之间的经济协作，推动煤炭、焦化、冶金、电力产业链条的有序衔接。加快信用担保、贷款公司等融资服务体系建设，多渠道解决企业融资难题。继续实施困难企业社会保险"五缓四降三补贴"政策，减免焦化企业政府性基金和收费，清理取消不合理行政收费项目，对重点企业封闭管理，坚决查处"三乱"行为。全面落实促进民营企业发展意见，支持我市民营企业家在本地投资创业，吸引从煤炭行业退出的资金投向鼓励类领域，实现二次创业。

　　努力扩大消费需求。继续落实国家家电、汽车、摩托车、农机下乡等鼓励消费的补贴政策，努力提高广大群众消费能力与消费意愿。着力扩大住房消费，增加中小户型商品房供应，抑制房价过快上涨。继续抓好"万村千乡市场工程"、"新网工程"、"放心粮油工程"，大力发展连锁经营、电子商务等新型流通方式。整顿和规范市场秩序，营造便利、安全、放心的消费环境。

　　各位代表，全力保持经济平稳较快增长，既是增强区域综合实力的需要，也是改善民生的基础。我们一定要抢抓机遇，克难攻坚，努力实现经济社会发展既定目标!

　　（二）优化产业结构，加快转变发展方式

　　金融危机表面上冲击的是经济发展速度，实质上检验的是经济结构和发展方式。后危机时代必须加快结构调整，大力发展低碳经济、循环经济、绿色经济，促进高碳经济向低碳经济、单一产业支撑向多元产业拉动、总量扩张向创新驱动、产业节点向产业链条、产品分散发展向产业集群发展转变。

　　整合提升优势产业。煤炭、焦化、冶金、电力、装备制造是我市的优势产业，必须以增强综合竞争优势为目标，实施信息化带动工业化，鼓励同行业上下游产业以资本为纽带，推进兼并重组，坚定不移走园区化、规模化、循环式发展道路。煤炭产业以建设大型现代化矿井为目标，全面推进129座资源整合煤矿改扩建工程，确保40%的矿井年内竣工投产，支持整合发展大型现代化洗煤、配煤企业，延伸煤炭产业链条；焦化产业以建设全省重要的焦化基地为目标，控制焦炭总量，延伸化产链条，着力建设洪洞、襄汾两个大型焦化园区和尧都、古县、安泽、乡宁4个焦化生产集中区，支持山焦、三维加快发展；冶金行业以发展特种钢、优质钢为目标，实施产能置换，调整优化布局，支持太钢等大型钢铁企业在我市实施兼并重组，扶持临钢做强做大，建设精品钢铁基地；电力产业以推进煤电一体化为目标，实施变输煤为输电战略，继续抓好4个在建项目，加快翼城、安泽、浮山、曲沃等电源点项目的前期工作；装备制造产业以发展系统集成和整机装配为目标，努力扩大零部件生产，支持平阳重工、汤荣汽配、华翔美的等重点企业规模发展，建设侯马、翼城、襄汾等装备制造基地。

　　培育壮大潜力产业。新型材料产业、高新技术产业、现代服务业极具发展潜力，要制定出台配套措施，促进规模化发展。材料产业充分利用我市丰富的石灰岩、石膏等矿产资源和煤矸石、粉煤灰等工业废料，大力开发新型建材、耐火材料、陶瓷材料等产品，加快推进蒲县、隰县干法水泥项目，努力建设新型材料基地；高新技术产业充分依托临汾、侯马开发区科技孵化中心的优势，积极引进科技含量高、市场前景广的项目。支持尧都区光宇LED电源、同世达光伏电源等企业扩大规模、聚集发展；现代物流业加快侯马国际化物流园区建设，扩大方略保税物流、宝特国际物流、北方轻工城等企业规模。整合搬迁临汾市区现有市场，加快琛士通、深国投购物中心、馨阳农副产品批发中心等项目建设，形成北城物流园区。积极发展金融、信息、咨询、科技、评估等现代服务业。

　　加快发展朝阳产业。依托文化底蕴深厚、旅游资源丰富的优势，大力发展文化旅游产业，实现由旅游资源大市向旅游强市转变。高起点做好旅游产业发展规划，鼓励民间资本参与旅游景区景点开发，建立多元投入机制，多渠道吸纳社会资金，加快经营体制创新。加大旅游景区景点硬件建设力度，促进吃住行游购娱等基础设施提档升级。实行全市旅游景点"一卡通"，构建区域旅游客源互换新机制。实施旅游包装推介，搞好旅游产品开发，叫响"中华根·黄河魂·尧乡韵"旅游品牌。深入推进"一带五区十点"建设，打造寻根祭祖、黄河风情、民俗民居、自然风光、红色旅游精品线路，形成旅游串点成线连片新格局。

　　各位代表，调整优化经济结构、促进发展方式转变，是一项长期艰巨的历史任务。我们一定要持之以恒调结构、常抓不懈促转型，务求取得扎扎实实的成效!

　　（三）加大支农力度，夯实"三农"发展基础

　　充分发挥临汾农业发展的比较优势，加大统筹城乡力度，以促进农民增收为目标，以农业产业化经营为方向，以改善农村发展条件为重点，扎实推进发展新农业、建设新农村、培育新农民"新三农"工程。

　　认真落实强农惠农政策。坚持"三个优先"要求，市县两级财政支农资金安排6.79亿元，增长33.8%。认真落实国家粮食直补、良种补贴、农机具购置补贴、农资综合补贴等政策，全面执行省政府各项扶持措施，实行各种补贴"一折通"制度，防止截留挪用。

　　推广以煤补农、以矿帮村的经验，落实企业支农捐赠优惠政策，扎实开展机关结对帮扶活动，形成全社会倾力支持"三农"工作的良好氛围。

　 大力发展新农业。实施以水兴农工程，完成五马水库、清峪水库、西里水电站大坝主体工程，实施汾西灌区节水配套工程、中小型水库除险加固工程，配合抓好禹门口引黄东扩工程。完成水土流失治理40万亩，生态修复80万亩，新建淤地坝50座，建设高标准示范农田2万亩，改造中低产田26万亩；实施粮食增产工程，抓好16个小麦万亩丰产方、30个玉米万亩丰产方和小杂粮示范点建设，新增机械化保护性耕作面积20万亩，确保粮食总产稳定在16亿公斤左右；实施现代农业示范工程，建设百万亩水果、百万亩蔬菜、百万亩干果、百万亩中药材等4个示范基地，扶持特色农产品规模发展，争创名优品牌；实施标准化园区建设工程，大力推广曲沃磨盘岭农业综合开发和洪洞西梁林下经济发展模式，力争每个县（市、区）新增1个千亩以上标准化示范园区，新建100个标准化养殖小区；实施"393"龙头企业扶持工程，培育省级企业3户、市级企业9户、县级企业31户，新发展农民专业合作社400个以上。切实抓好农产品质量安全和动植物疫病防控。

　　加快建设新农村。按照因地制宜、分类指导的原则，鼓励村级组织发挥优势，量力而行，按规划推进新农村建设。要在巩固提升原有试点村和重点推进村的基础上，集中力量抓好新确定的236个重点推进村建设。着力实施新农村建设"325"精品工程，树立30个标兵村，打造20个连片区，培育50个亮点村。全面完成"五个全覆盖"任务。搞好移民搬迁和沼气扶贫，实现扶贫开发由整村推进向片区开发迈进，统筹搞好外资扶贫项目实施，新增沼气用户1.5万户。大力整治乡村环境卫生，积极开展"和谐乡村"创建，全面提升新农村建设水平。

　　 积极培育新农民。以培育新型产业农民为目标，开展多层次、多渠道、多形式的培训，完成农村实用人才培训12万人。以培训新型务工农民为目标，完成农民职业技能培训2万人。健全农民工劳务输出服务机构，加强与劳务输入地区的联系，为农民工外出务工搭建平台，转移劳动力4.万人。深化户籍制度改革，降低农民进城门槛，促使农村富余劳动力向非农产业和城镇转移。继续搭建信息、技术和销售平台，促进农民增收。围绕"乡风文明"建设，加强移风易俗教育，开展科技、文化、卫生"三下乡"活动。出台农民工返乡创业优惠政策，提供信息、资金、技术等方面的服务。

　　 各位代表，"三农"问题事关全市300万农民的切身利益，始终是我市工作的重中之重。我们一定要带着感情抓"三农"，扎扎实实抓"三农"，千方百计推动"三农"工作迈上新的台阶！

　　（四）加快城市建设，提高城镇化水平

　　城市支撑着经济发展，展示着地方形象。我们要把城市建设作为战略重点，突出完善城市功能，全面强化城市管理，努力提高城市品位。

　　 加紧编制城镇规划。按照"政府组织、专家领衔、部门协作、公众参与、科学决策"的原则，加快编制城镇规划。围绕打造"唐尧文明圣地、新兴工业之都、和谐宜居新城"的城市定位，加快编制《河西新城控规》、《南部新城控规》、《空港园区控规》、《高铁车站地段城市设计》等6个控制性详细规划，完成《城中村改造规划》、《城市燃气专项规划》、《城市排水、污水处理与中水回用专项规划》等10个配套规划的编制工作。加大县（市、区）总规、近期建设规划的编制力度，确保城市建设有规可依、有章可循。出台城乡规划管理办法和违法建设查处办法，加强规划执法，维护规划的严肃性和权威性。

　　 推动城市基础设施建设。临汾市区建设按照"拉大框架、拓展空间、建设精品、提升品位"的总体要求，集中力量打造河西新区，启动实施南北大道、锣鼓大桥西延、西关大桥西延、漪汾花园南路、新高中南路等重点工程。抓好东城新区文化、教育等公共服务设施建设。制定出台优惠政策，用市场化手段加大旧城改造力度，启动实施向阳路、北外环立交桥及道路改造工程，完成五一路立交桥建设，组团式改造一批城中村。按照"特色兴县、产业兴镇、园区带动"的思路，加快县城和重点建制镇建设。树立经营城市理念，建立和完善土地一级市场，加强土地收储，规范土地出让，盘活城市存量资产。出台城市土地管理实施意见，走出一条具有临汾特色的以城建城、以城养城的城市发展之路。

　　 创新城市管理体制。以降低管理成本、提高管理效能为目标，继续改革城市管理体制。按照建管分离、管养分离的原则，积极推进城市管理市场化进程。建立和完善市、区、街道、社区四级管理体制，构建目标明确、责任清晰、管理科学、考核严格的城市管理机制；建立和完善规划、土地、建设三个执法一体化协调机制，严格规范建设秩序，严查违法违规建设和失职渎职行为；建立和完善市容、交警、环卫三个管理一体化协调机制，不断提高城市管理水平。加强市民城市意识、交通意识、环保意识、卫生意识教育，提高市民文明素质，共同建设美好家园。

　　 各位代表，建设现代化城市、加快城镇化进程，既是提升区域竞争力的迫切要求，也是广大人民群众的热切期盼。我们一定要科学规划，打造精品，加强管理，真正把城市建设成为人民群众宜居宜业的乐园!

　　（五）推进生态建设，改善环境质量

　　生态环境问题仍然是临汾可持续发展的短板，直接影响着人民群众幸福指数的提高。必须毫不动摇促节能，坚持不懈治污染，打好节能减排攻坚战和持久战，促进生态环境持续改善。

　　 强力推进节能降耗。制定全市低碳经济发展规划，严格落实节能工作目标责任制，建立健全考核评价制度。依法加强节能管理，严格实行新上项目节能评估制度，认真落实差别电价、阶梯水价制度，加强重点能耗企业节能合同约束。大力推广节能技术，突出抓好135户工业企业的节能示范工作。推进废渣、废气、废水利用和生活垃圾资源化利用。加快建筑节能改造，新建建筑执行国家节能标准。全面拓展节能领域，倡导全社会低碳生产、低碳生活、低碳消费的新风尚。

　　 坚决整治环境污染。全面整治工业污染，坚决淘汰落后生产工艺和设施，严格执行环境影响评价和"三同时"制度。强化企业排污监管，所有国控、省控工业污染源全部实现在线监控。加大汾河、沁河等流域水污染防治力度，建立水质监测考核奖惩机制。以治理功能性污染为重点，加快环保基础设施建设，年底所有县（市、区）污水处理厂建成并投入正常运行，11个县市垃圾无害化处理场开工建设，吉县、大宁、永和要投入使用。临汾市区新增集中供热面积200万平方米，覆盖率达到90%以上。加快实施天然气接入工程，临汾市区城中村基本用上清洁能源。采取综合措施，抑制城市二次扬尘和汽车尾气。全面加强农村环境保护，推进生态文明村、环境优美乡镇建设。确保各县（市、区）二级以上天气达到310天以上，市区大气综合污染指数稳控在1.8以下。

　　 大力实施生态建设。坚持山上治本与身边增绿同步推进，搞好国家重点林业工程建设，大力实施通道绿化、交通沿线荒山绿化等10大林业工程，积极发展碳汇林业，完成造林50万亩、四旁植树1200万株。加大城市绿化力度，确保城市建成区绿化覆盖率提高1个百分点以上，达到省级园林城市标准。加强龙祠水源地环境保护，推进塔儿山、二峰山生态综合治理，抓好霍州棚户区改造和沉陷区治理工程。加快汾河城区段生态工程建设，展现"一川清水、两岸锦绣"的靓丽景观。

　　 各位代表，建设生态文明、提升环境质量，是构建和谐社会的重要内容。只要我们万众一心齐参战，齐心协力合攻坚，就一定能够建成山川秀美、碧水蓝天的新临汾!

　　(六)加强安全生产，提升安全发展水平

　　安全始终是临汾时刻不能松懈的重要工作，忽视了安全就等于放弃了发展。要把保障人民群众的生命权、财产权作为各级政府的重大政治责任，切实强化措施，狠抓落实，巩固来之不易的良好局面。

　　 切实增强安全意识。牢固树立"抓安全就是抓发展、不安全就不能大发展"的理念，把安全发展放在政府工作的重要位置。认真落实"安全第一、预防为主"的方针，真正做到思想上高度重视，工作上常抓不懈。强化"隐患即事故"的意识，做到关口前移、重心下移。加强安全教育，增强全社会安全意识，从思想上筑起安全生产的牢固防线。

　　 严格落实安全责任。重点落实各级政府的领导责任、部门监管责任和企业主体责任。按照属地管理原则，认真落实行政首长负责制，班子其他成员按照"一岗双责"抓好分管领域的安全工作，确保各行各业的安全生产事事有人抓、时时有人管，不留空当，没有死角；强化部门监管责任，做到制度严格可行、检查严密细致、责任追究严肃；强化企业主体责任落实，扎实开展安全质量标准化建设，引导企业加大安全投入，健全管理制度，严格生产和作业现场管理，落实关键环节和重要岗位的责任。严格安全工作考核奖惩，坚决落实"一票否决"制度。

　　 扎实推进专项整治。突出抓好煤矿、非 煤矿山、尾矿库、冶金、道路交通、消防、森林防火等重点行业和领域的安全工作，加强加气站、加油站等重大危险源和人员聚集场所、地质灾害易发区的防范监控。采取严格有效的措施，加强隐患排查治理。开展"春雷"行动，对非法违法生产保持高压态势。煤矿安全重点抓好过渡期矿井和"5+3"主体企业之外的矿井安全管理工作，明确5人监管小组职责，更加注重矿井安全预防和治本，更加注重对煤矿"一通三防"的监督检查，更加注重长效机制建设，确保全市煤炭百万吨死亡率等四项指标控制在全省平均水平以下。

　　 各位代表，安全生产人命关天，安全责任重如泰山。我们一定要铭记教训，痛定思痛，毫不松懈地抓好安全生产，为经济社会平稳较快发展创造良好的环境!

　　（七）坚持改革开放，增强经济社会发展活力

　　改革是发展的原动力，开放是发展的助推器。应对挑战需要改革，加快发展必须开放。

　　 抓好关键环节改革。科学编制"十二五"发展规划。全面完成市县两级政府机构改革，适时启动乡镇机构改革，稳妥推进事业单位分类改革。加快推进国企改革，完成市属5户国有企业政策性破产的扫尾工作。严格规范国有资产管理，坚决防止国有资产流失。继续深化财税体制改革，完善财政资金管理，优化财政支出结构。加强煤焦领域税费征缴管理，确保应收尽收。扎实推进集体林权制度、医药卫生体制、文化体制、投融资体制等方面改革。

　　 努力扩大对外开放。积极参加"高交会"、"中博会"、"能源博览会"等招商引资活动，精心组织在上海、青岛、香港、台湾等地举办招商项目推介会。拓宽招商引资渠道，鼓励民间组织、企业、商会招商，支持临汾籍在外人士回乡投资，在引进资金、引进项目、引进技术、引进人才等方面实现重大突破。继续加强与友好城市、友好地区合作，拓展合作领域。积极扩大对外贸易，巩固欧美传统市场，开拓东盟、南美、俄罗斯等新兴市场。

　　 加大科技创新力度。继续搭建产学研合作平台，推进主导产业技术进步和科技创新。采取奖励扶持措施，鼓励引导企业加大研发投入，引进科技人才，建立研发中心，提升企业创新能力，加快临汾、侯马两个开发区科技孵化中心建设。围绕农产品升级换代，培育10个农业产业化科技示范基地。开展科技兴县活动，力争两个县成为全国科技富民强县试点县。

　　 （八）着力改善民生，促进社会和谐

　　保障和改善民生是政府义不容辞的责任。我们要加快发展民生和社会事业，优先解决人民群众关注的现实问题。

　　 全面振兴教育事业。继续加大教育投入，市县两级教育预算支出安排21.5亿元，增长31%。积极推进教育资源整合，优化学校布局，促进教育公平。加快临汾十三中、临汾二中、东城小学建设，缓解市区中小学班容量过大的问题。加快教育改革，端正办学方向，推进素质教育。高度重视基础教育，加强教师队伍建设和教学管理，提升教学质量。规范民办教育，壮大职业教育。办好职业学院、临汾学院和广播电视大学，支持山西师大建设发展。完成教育规划制定，科学指导教育事业振兴。

　　 提高医疗卫生水平。继续实施"县提升、乡达标、村覆盖"工程，着力改善医疗条件。完善新型农村合作医疗制度，提高财政补助标准。扎实做好市第四人民医院和古县人民医院公立医院改革试点工作，完成22个城市社区卫生服务站设备配备任务。加强疾病预防控制、妇幼保健和食品药品监督管理。大力发展中医药事业，恢复市中医医院机构。继续稳定低生育水平，实施"优生促进工程"。

　　 健全社会保障体系。积极推进城镇企业各类职工参加养老、失业、医疗和工伤保险，扩大城镇居民基本养老、基本医疗覆盖面。扎实做好农村社会养老保险工作，覆盖人数达到24.3万人。抓好城乡低保规范化管理，提高五保对象集中供养水平，完善临时救助制度，妥善解决农村居民生活和取暖用煤问题。为市区合同制清扫清运工人购买社会保险，解除城市环卫工人后顾之忧。

　　 全力促进创业就业。认真落实各项促进创业就业的扶持政策，鼓励发展劳动密集型企业、中小企业和服务业，完善政府投资项目优先安排当地劳动力机制，努力增加就业岗位。重视做好大学生就业和城镇退役士兵安置工作。加大公益性岗位开发力度，积极解决零就业家庭成员、"4050"人员、大龄就业困难人员就业难问题，千方百计实现新增城镇就业4.6万人。

　　 实施住房安居工程。开工建设经济适用房9.6万平方米、1600套，廉租房8.2万平方米、1640套，城市棚户区改造建设住房5.35万平方米、575套，国有工矿棚户区改造新建住房4.56万平方米、638套。完成漪汾花园经济适用房建设，为近3000户中低收入家庭提供住房保障，使1000户居民按期入住。完成农村危房改造3633户，为500户农村贫困残疾人进行危房改造。出台经济适用房管理办法和廉租房保障办法，确保困难群体住有所居。

　　 努力促进文化繁荣。加快发展文化事业，启动实施59个文化站、294个农民书屋建设，积极开展送戏、送电影进农村、进社区、进企业。大力发展文化产业，扶持蒲剧、眉户 等传统艺术，努力打造精品剧目；深入挖掘和保护非物质文化遗产，支持威风锣鼓、木版年画、剪纸等民间艺术走向市场。加强未成年人思想道德教育，强化网吧等文化市场管理，坚决扫除"黄赌毒"等社会丑恶现象。大力开展全民健身活动，提高竞技体育水平。树立文明新风，引深"爱我临汾、树我形象、建我家乡"活动。

　　 着力强化社会管理。坚持堵疏结合，正确处理各种社会矛盾，坚决查处纠正城市拆迁、国企改制、土地征用、环境保护等方面损害群众利益的各种问题。完善信访工作机制，做好人民调解工作。规范劳动用工管理，维护劳动者合法权益。加强社会治安综合治理，大力弘扬见义勇为，严厉打击各类刑事犯罪活动，确保社会稳定。切实提高防震减灾能力，不断发展人民防空事业。完善应急管理体系，预防和妥善处置各类突发事件。扎实做好第六次人口普查工作。认真落实民族宗教政策，依法管理宗教事务，保护正常的宗教活动。

　　 加强民主法制建设。自觉接受人大及其常委会的法律监督、工作监督和人民政协的民主监督，严格执行市人大及其常委会决议、决定，加强与各民主党派、工商联、无党派人士和人民团体的联系，认真办理人大代表、政协委员的议案、提案。充分发挥工会、共青团、妇联、科协、侨联等群团组织作用。支持人民法院、检察院依法履行职责。努力扩大基层民主，保障人民群众的知情权、参与权、监督权。全面落实"五五"普法规划，扎实推进"法治临汾"建设。高度重视国防后备力量建设，广泛开展"双拥"活动，做好民兵预备役工作。

　　 各位代表，保障和改善民生，重在办实事。今年，市政府向全市人民承诺办好10件实事：

　　1、完成126.3万平方米中小学校舍改造任务；2、全面完成村级卫生室建设任务，在60%的政府性举办的基层医疗卫生机构实行基本药物制度；3、对3000名就业困难人员进行就业援助，对300名贫困残疾儿童实施康复援助，新建和改扩建农村敬老院17所；4、解决农村42万人饮水不安全问题；5、贯通全长57公里的市区内环一级路；6、实施污水处理工程，实现市区污水零排放；7、汾河生态公园和古城遗址公园向市民开放；8、建设和完善1180个粮油经销点，新建200家连锁农家店；9、完成6500人移民搬迁任务；10、开工建设市文体中心。以上10件实事，涉及人民生活诸多方面，我们就是要把关乎民生的事情，挂在心上，抓在手上，力争为全市人民交上一份满意的答卷!

　　三、加强政府自身建设

　　推动负重转型、促进绿色崛起、加快统筹城乡、实现科学发展，政府自身建设是关键。我们将以建设人民满意的政府为目标，继续推进学习型政府、服务型政府、法治型政府、廉洁型政府建设，努力提高领导科学发展的能力和水平。

　　 (一)加强学习，增强科学发展的坚定性。

　　 新形势要求我们重视学习，新任务要求我们坚持学习，新挑战要求我们深化学习。我们要加强政治理论学习，端正从政理念；加强行政管理和法律知识学习，提高依法执政水平；加强市场经济知识学习，提高驾驭经济工作的能力；注重向实践学习，向群众学习，提高真抓实干、处置复杂问题的本领。坚持知行合一，学以致用，用科学的理念谋划政府工作，用科学的态度解决疑难问题，用科学的方法应对困难挑战，通过学习，坚定信心，通过学习，推动创新。

　　 (二)转变职能，提高执行落实的实效性。

　　 明确政府角色定位，理顺政府与企业、市场、社会中介组织的关系，着力搞好经济调节和市场监管，突出加强社会管理和公共服务，更加重视发展民生等社会事业，把经济工作的重点转移到为市场主体服务和创优发展环境上来。落实政府工作责任，健全目标责任制，强化执行、反馈、监督、考核。实行行政问责，严格奖惩兑现，把定下的事情落到实处。提高政府工作效率，继续推进行政审批制度改革，加快电子政务建设，实行首办负责制、限时办结制。强化行政效能监察，真正解决门难进、脸难看、话难听、事难办的问题。切实减少会议，努力压缩文件，腾出工作精力谋大事、办实事。

　　 (三)依法行政，确保决策施政的规范性。

　　 全面推进法治政府建设，坚持依法行使职权，依法履行职责。健全政府议事规则，坚持依法决策、科学决策、民主决策，发挥法律顾问、专家学者、社会听证在决策中的作用。强化行政监督，大力推进政府信息公开，完善信息和新闻发布制度，广泛接受人民群众和新闻媒体监督。加强行政执法监督，着力解决一些执法领域落实不下去、严格不起来的问题，查处徇情枉法、徇私枉法、贪赃枉法行为，真正做到有权必有责、用权受监督、侵权应赔偿、违法要追究。

　　 (四)清正廉洁，强化拒腐防变的自觉性。

　　 全面落实政府系统党风廉政建设责任制，规范公务人员从政行为。加强行政监察，坚决查处违纪违法案件。深入开展廉洁从政警示教育，防止权力寻租和行政腐败，纠正政府权力部门化、部门权力个人化、个人权力利益化行为。继续引深煤焦领域反腐败专项斗争，加大治理商业贿赂力度。强化政府投资项目审计，规范工程招投标、财政转移支付、土地资源使用、政府采购、国有资产转让等行为。厉行勤俭节约，制止铺张浪费，狠刹奢靡之风，以廉洁从政、清廉为民的良好形象取信于民!

　　各位代表，做好今年的工作，任务艰巨而繁重，责任重大而光荣。让我们在中共临汾市委的坚强领导下，坚持以邓小平理论和"三个代表"重要思想为指导，深入贯彻落实科学发展观，同心同德，群策群力，扎实工作，开拓创新，加快建设经济繁荣、社会和谐、民生殷实、环境优美的新临汾，以优异的成绩迎接建市10周年!
